# Notes on the Origin of Modern Dentistry for the Laity

**Published:** 1921-04

**Authors:** Alfred A. Crocker


### Notes on the Origin of Modern Dentistry for the Laity

By ALFRED A. CROCKER

THE advertising by tooth preparation manufacturers has had the tendency to leave the impression on a great majority of the public that a tooth brush and a package of paste will preserve the teeth. In large type most pastes are heralded as tooth savers—and by association of ideas health preservers—and in small type the advertisements go on to say, "See your dentist semi-annually," which is indeed very kind to the profession.

The first advertising publicity given dentists as a profession ethically is possibly in the expression "See your dentist," or "See your dentist periodically" used by the tooth preparation manufacturers. Although this from an advertiser's point of view might have been only to pay off the profession for recommending a certain tooth paste, during my interviews with dentists I discovered that the dentist did not object, and in fact many liked it. My deduction was that they certainly would not then object if the profession received the entire mention, or the tooth paste only incidental mention.

Dentistry was named as essential and many a recruit found out how essential it was when he received his notice of dental requirement and had to go to a dentist before he could be mustered in. This in itself was a complete demonstration of the possibilities of dental propaganda and pointed out to me one way it could be done. And the time had come when it could most easily be accomplished, for the word "preparedness" had assumed a larger meaning by including dentistry. The best known reason for mentioning dentistry for medical examination of recruits for the Army is that the subject of group diagnosis is one that had held the attention of the medical world for recent years and had about reached its zenith of insistent indication as a universal necessary move when the war broke out. This, together with the results of the dentists finding by dental research on focal infection and value of preventive dentistry brought dentistry prominently to the front. Besides a uniform ration as supplied to the Army requires a uniform predigestive apparatus and indicated a stated requirement of sufficient teeth on the part of applicant to derive sufficient nourishment from the ration for active engagement in war.

"Industrial Dentistry" has been the means of reaching a great number of people with the gospel of dentistry in both the laboring and capital classes, and still forms one of the most effective methods of reaching large numbers of people. It is a business proposition and safety welfare work of the highest order, completing the hospital which is incomplete without it.

As a selling talk to corporation managers who through their medical departments are urged to install dental equipment for safety tooth work I would say that the principal benefits to a corporation from dentistry in the factory are derived through health maintenance. This has its effect on reduction of turnover of labor and decrease of hours lost from production; in other words, increase of production. Dentistry is no more welfare work than is medical care. Both are safety work and that is different, for this means a benefit first to the labor element which is for the purpose of maintenance of health for production. In each plant the idea is maintenance of production for the specific out-put that it manufactures. As it is known that the cost of hiring and teaching the initial steps in the work and dismissal of each employee is about $60, the reduction of turnover is valued at the rate of $60 per person. In large factories the reduction is in figures which make the work pay splendidly with a big margin over the cost of the work involved in the entire medical department of which the dental service is a part. Mutual aid and safety welfare in operation are almost identical. If the employees pay for it, it is mutual aid; if the employer pays for it, it is safety welfare work. Plain welfare work does not exist in factories for that expression applies in community work and so-called welfare centers. Safety welfare is human maintenance and efficiency work along scientific lines for the benefit of production. Plain welfare work is relief of suffering and care of the needy for humanity's sake. When feeling exists over safety work it is due to a misunderstanding of the difference between safety welfare and plain welfare. Besides, another difference is that safety welfare merely indicates what ought to be done and welfare actually does relief work.

To go a little further with this calculation of the money value of the saving by reduction of turnover. On an operative force of one thousand a turnover of one hundred and fifty percent was experienced in one factory doing the same class of work that another with two hundred percent turnover. One had medical and dental service and the other had not. According to the unit saving of $60 for individuals the saving to the company having medical and dental service on five hundred men was $30,000.00. If we estimated that the dental phase of this work was one-fifth, the dental department saved $6,000. In the same factory the hours saved by the dental department was two thousand, giving each hour saved a sales value of two dollars, this makes $4,000 saved; together these two items total $10,000 as a credit for the dental service. While these figures are not exact, they give a basis on which a calculation of the value of dental service to a factory can be made.

Besides those already mentioned in the main article on "Modern Dentistry for the Laity," after being tried out in different territories to the satisfaction of all concerned the most recent development is from Boston, where a well-known advertising firm, seeing that the approach of the subject is from the accepted ethical publicity point of view and merely the application of present-day advertising methods to known truths about dentistry, have undertaken an intensive campaign of ten weeks in newspapers. This shows the correctness of this analysis when viewed from the expert publicity man's point of view.

The National Safety Council has incorporated dental service and dental prophylaxis in their regular schedule for safety control. All this has taken place since 1917. Safety in the factory nowadays does not mean merely prevention from accidental injury, it means also prevention from injury that eventually comes to those not informed on various forces that are bound to affect their welfare if not pointed out by an overseeing superintendent or service director, who has at his command specialists in various professions and sciences making periodical examinations. Safety is the purpose of all welfare work and this now very properly includes preventive dentistry. Among the many activities of the National Safety Council, those of the Health Service Section have always played a prominent part. This Section was formally organized by the Fourth Annual Congress of the National Safety Council, Philadelphia, and its record has been one of constantly increasing usefulness and accomplishment. The term "Health Service" is a most comprehensive one. Some of the activities it symbolizes are as follows:

(a) **Sanitation:** 1. Illumination. 2. Ventilation. 3. Personal comfort—water supply, toilets, washing facilities. 4. Infectious Diseases—occupational, dust, poisonous fumes and gases, unsafe practices. 5. Eye protection.

(b) **Physical Examination.**

(c) **Surgical-Medical Supervision and Care:** 1. Equipment staff technic scope. 2. Records, statistics. 3. Dental care.

(d) **Health Hazards:** 1. Statistics of morbidity and mortality. 2. Mutual insurance. 3. Methods of health education—shop bulletins, home leaflets. 4. Mutual betterment—lunch room, food, alcohol, building and loan association. 5. Home environment—housing, sanitation, visiting nurse.

This shows that dental service is permanently established as an aid to production.

